# DMP1-mediated transformation of DPSCs to CD31^+^/CD144^+^ cells demonstrate endothelial phenotype both *in vitro* and *in vivo*

**DOI:** 10.3389/fcell.2025.1630129

**Published:** 2025-08-08

**Authors:** Amudha Ganapathy, Yinghua Chen, Velavan Bakthavachalam, Anne George

**Affiliations:** Brodie Tooth Development Genetics & Regenerative Medicine Research Laboratory, Department of Oral Biology, University of Illinois at Chicago, Chicago, IL, United States

**Keywords:** dental pulp stem cells, dentin matrix protein 1, human umbilical vein endothelial cells, endothelial cells, endothelial cell phenotype, angiogenesis

## Abstract

**Introduction:**

Dental pulp stem cells (DPSCs), can differentiate into endothelial cells (ECs), offering a promising strategy for generation of new blood vessels which is crucial for tissue repair and regeneration. Many studies have focused on optimizing conditions for differentiating DPSCs into ECs *in vitro* and subsequent validation of the vasculogenic potential of newly generated ECs *in vivo*. Previously, we demonstrated the ability of the HUVEC ECM scaffold along with DMP1 stimulation would drive endothelial-specific lineage of DPSCs.

**Methods:**

In this study, DMP1-treated DPSCs were cultured on HUVEC ECM for 7 days and sorted using angiogenic-specific markers CD31 and CD144. The cells were separated into a positive fraction (CD31^+^/CD144^+^) and a negative fraction (CD31^−^/CD144^−^). To assess if ECs transformed from DMP1 stimulated DPSCs maintain their endothelial properties over time, we cultured both the positive CD31^+^/CD144^+^ and negative CD31^−^/CD144^−^ fractions along with unstimulated DPSCs and assessed their angiogenic characteristics by gene expression analysis, functional properties using a tubule formation assay and *in vivo* subcutaneous implantation model.

**Results and discussion:**

The findings of this study indicate that the CD31^+^/CD144^+^ fraction, retains both the phenotypic and functional characteristics of ECs, in contrast to the CD31^−^/CD144^−^ fraction. Furthermore, *in vivo* analysis of the sorted ECs using the subcutaneous implantation model exhibited neovascularization along with the expression of vasculogenic markers. Overall, DPSC-derived ECs obtained by stimulation with DMP1 and cultured on HUVEC-ECM function as typical vascular ECs. This strategy, could be exploited for the development of vasculogeneis and as a therapeutic potential for tissue repair and regeneration.

## Graphical Abstract

**Figure:**
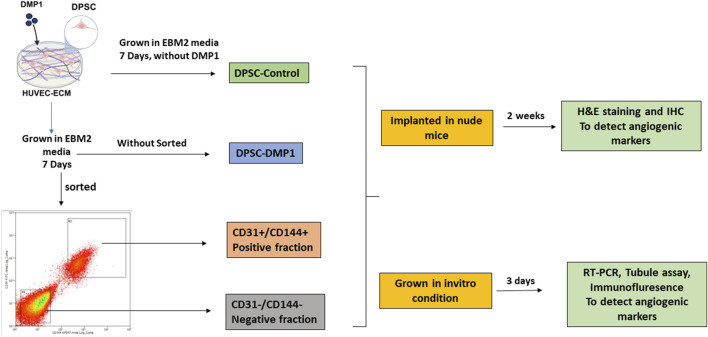
Schematic overview of the experimental approach.

## Introduction

Vascularization is one of the major challenges that hinders the clinical use of engineered tissues. During the development and regeneration of dentin, a critical issue is cell viability maintenance and promoting the differentiation of preodontoblasts. This process relies on having a functional vascular system. Blood vessels not only supply oxygen and nutrients, transport ions, and remove metabolic waste; they also facilitate the recruitment of progenitor stem cells and immune cells. Neovasculogenesis is a critical process in wound healing (Moreira et al., 2022) and plays a vital role in bone tissue engineering by supporting vascularization. This process is essential for tissue repair and regeneration, as it ensures an adequate blood supply to maintain the viability of engineered tissues (Yang et al., 2020; Masson-Meyers and Tayebi, 2021). In the absence of a functional network, implanted cells may undergo apoptosis if they do not receive enough oxygen and nutrients. Therefore, there is a clinical need to develop *de novo* vascularization therapeutics for the regeneration of the dentin-pulp complex and to support various tissue engineering applications.

Neural crest-derived dental pulp somatic cells have shown significant potential in contributing to the formation of vascular structures for tissue regeneration. (Luo et al., 2018). Certain mesenchymal stem cells promote therapeutic angiogenesis by releasing angiogenic growth factors and differentiating into endothelial cells (ECs) (Tao et al., 2016; Mohamad Yusoff and Higashi, 2023). Dental pulp stem cells (DPSCs) stand out among other stem cell types due to their strong ability to stimulate angiogenesis (Luzuriaga et al., 2020). Research shows that DPSCs release several angiogenic factors, including interleukin-8, angiogenin, endothelin-1, angiopoietin, and insulin-like growth factor binding protein-3 (Kato et al., 2020; Zhang et al., 2022). Furthermore, they secrete vital signaling molecules such as Vascular endothelial growth factor A (VEGFA), Platelet-derived growth factor (PDGF), Basic fibroblast growth factor (bFGF), and Nerve Growth Factor (NGF) which are essential for the survival and proliferation of vascular ECs (Tsutsui, 2020; Su et al., 2025). Additionally, these signaling molecules promote endothelial tubulogenesis, which is an essential step in the development of new blood vessels. (Lin et al., 2024). In animal models of diseases such as myocardial infarction, ischemia, or neurological disorders. DPSCs have shown therapeutic promise. They improve vascular function and promote neovascularization (Luo et al., 2018; Mattei et al., 2021). Moreover, DPSCs can be differentiated into endothelial like cells. When exposed to VEGFA, DPSCs express increased level of markers such as VEGFR1, VEGFR2, von Willebrand factor, which indicates their potential differentiation into ECs (Mullins et al., 2019). Even in their undifferentiated state, DPSCs express VEGFR1, which makes them more likely to become ECs compared to mesenchymal stem cells from bone marrow or adipose tissue (Luo et al., 2018; Janebodin et al., 2021; Mattei et al., 2021). Furthermore, ECs derived from DPSCs express VE-Cadherin, an essential adhesion molecule that aids in the integration of DPSC-derived microvessels with host blood vessels (Sasaki et al., 2020). Recently, researchers have used DPSCs to 3D print tissue that resemble the dentin pulp complex with integrated blood vessels, demonstrates a new strategy for customized tissue regeneration (Hilkens et al., 2017; Quigley et al., 2024). Additionally, DPSCs have are multipotency multilineage and can differentiate into various cell types, including vascular ECs, odontoblasts, osteoblasts, adipocytes, and chondrocytes (Gronthos et al., 2000; Aydin and Şahin, 2019; Bai et al., 2023). Due to their low immunogenicity and capacity to foster immune tolerance, these cells are ideal for tissue engineering and cell-based treatments. Endothelial colony-forming cells (ECFCs) are a potential source of stem cells, but their unipotent nature limits their application primarily due to blood vessel repair. (Melero-Martin, 2022; Chambers et al., 2025). Also, ECFCs are hard to collect in large amounts from peripheral blood. In contrast, DPSCs are more accessible and versatile, offering wider regenerative capabilities. The growing evidence highlights DPSCs as a promising cell source for regenerative medicine and tissue engineering due to their angiogenic potential, ability to differentiate, immunological benefits, and ease of collection. Research published studies (Gong et al., 2017) showed that stem cells from exfoliated deciduous teeth and DPSCs could be differentiated into ECs by the de-cellularized matrix of Human Umbilical Vein Endothelial Cells (HUVECs), however, their low efficiency of endothelial differentiation restricts their translational therapeutic applications. Therefore, we have decided to use biological cues from the HUVEC-Extracellular Matrix (HUVEC-ECM) along with DMP1 to enhance the differentiation potential of adult stem cells into ECs. Dentin matrix protein 1 (DMP1) is a bone and tooth-specific noncollagenous ECM protein initially identified from the dentin matrix (George et al., 1993). During mineralization, the crystal nucleation and growth processes are initiated by DMP1. During development, the initial expression of DMP1 coincides with bone and dentin mineralization, indicating that DMP1 is actively involved in regulating the temporal and spatial aspects of mineral nucleation (Hao et al., 2004; He and George, 2004). Apart from its role in mineralization, DMP1 can function as a signaling molecule and promote osteoblast and odontoblast differentiation at several stages of development (Ravindran and George, 2014). The development of bones and teeth depends profoundly on vasculogenesis. Due to their coordinated and interdependent occurrence, matrix mineralization and bone healing are closely related processes (Grosso et al., 2017; Diomede et al., 2022). We recently reported an *in vitro* differentiation method using a combination of rDMP1 stimulus and HUVEC-ECM scaffold, and demonstrated improved transformation of DPSCs into ECs (Ganapathy et al., 2024). In this study, we sorted the endothelial-like cells using CD31 and CD144 as angiogenic specific sorting markers. This combination offers greater specificity for identifying ECs and lowers the risk of including other cell types in the sorted group. Next, we characterized and evaluated the vasculogenic potential of the sorted ECs both *in vitro* and in an *in vivo* model of the subcutaneous implantation assay. Overall, the promising findings from this study reveal the ability of DMP1, in association with HUVEC-ECM, to enhance the conversion of DPSCs into endothelial-like cells, which progressively exhibit the functional characteristics of true ECs over time while retaining their original identity.

## Materials and methods

### Cell culture

Human dental pulp stem cells were a kind gift from Dr. Songtao Shi at the University of Pennsylvania (Gronthos et al., 2000), and they were isolated in accordance with IRB protocol number 816238, which was established by the National Institutes of Health Office of Human Subjects Research. The cells were cultured in α-minimum Eagle’s medium (Corning) containing 20% of fetal bovine serum (FBS; Thermo Fisher Scientific) with 1% antibiotic-antimycotic, which contains 25 μg/mL of Gibco Amphotericin B (Thermo Fisher Scientific), 10,000 units/mL of penicillin, and 10,000 μg/mL of streptomycin. This culture was maintained at 37°C in a humidified CO2 incubator. When the cells reached about 80% confluency, they were divided at a 1:3 ratio (one passage). In every experiment, only cells from passages three through six were used. Experimental approach overview depicted in the Graphical Abstract.

### Differentiation of DPSCs on HUVEC-ECM

DPSCs were seeded at a density of 0.3 × 10^6^ cells per well onto plates coated with HUVEC-ECM and cultured in EBM-2 media supplemented with 10% FBS. The rDMP1 at a concentration of (500 ng/mL) (Eapen et al., 2010) was added to the media. The cells were fed with freshly prepared media every other day.

### Cell sorting by FACS

After 7 days of culture in HUVEC-ECM, supplemented with DMP1 in EBM2 media, DPSCs were harvested at a density of 10 × 10^6^ cells per FACS tube. Following a PBS wash, the cells were incubated with 15 µL of anti-human CD31 and anti-human CD144 antibodies for 30 min at room temperature in a dark environment. After being incubated, the cells were resuspended in stain buffer and given another PBS wash. A Moflo Astrios EQ device (UIC, RRC facility) was then used to sort the cells according to the expression of CD31^+^ and CD144^+^. ECs were represented by the positive fraction CD31^+^/CD144^+^, while cells lacking both CD31^+^ and CD144^+^ expression were represented by the negative fraction CD31^−^/CD144^−^. Both *in vitro* and *in vivo* animal studies were conducted using the sorted and unsorted DPSCs.

### *In vivo* animal studies

Four groups of DPSC-derived cells were used for implantation experiments: Group 1 consisted of unsorted DPSCs cultured in HUVEC-ECM alone (unsort-control); Group 2 included unsorted DPSCs cultured in HUVEC-ECM with DMP1 (unsort-DMP1); Group 3 represented the CD31^+^/CD144^+^ positive fraction; and Group 4 represented the CD31^−^/CD144^−^ negative fraction. Groups 3 and 4 were isolated from Group 2 via antibody-based sorting using CD31 and CD144 markers. A total of 5 × 10^5^ cells from each group were seeded into collagen scaffolds (RegenePro Plug, Syntacoll GmbH) and incubated for 12 h. Next, 8-week-old athymic nude mice (Jackson Laboratory) had their backs subcutaneously implanted with the cell-seeded embedded scaffolds. Mice were sacrificed 2 weeks after implantation, and the scaffolds were taken out, preserved for 12 h at 4°C in 4% neutral buffered formalin, dehydrated using graded ethanol, and embedded in paraffin. A microtome (HistoCore Biocut, Leica) was used to prepare sections (5 μm thick) for histological examination. All animal procedures were conducted under a protocol approved by the UIC Animal Care Committee (ACC: 21-174).

### Immunohistochemical analysis

After tissue sections were deparaffinized, antigen retrieval was carried out for 30 min at 95°C in Citric Acid Buffer (10 mM Citric Acid, 0.05% Tween 20, pH 6.0). Following several PBS washes, the sections were treated with 0.25% Triton X-100 in PBS for 30 min, and then they were rinsed with PBS. Following a one-hour block with 10% BSA in PBS at room temperature, the sections were incubated with 1:100 rabbit anti-human CD31 (ab28364, Abcam, Cambridge, MA), ERG (EPR3863, Abcam, Cambridge, MA), VEGFA (ab46154, Abcam, Cambridge, MA), VE-Cadherin (ab33168, Abcam, Cambridge, MA), HIF-1 (AB1, Abcam, Cambridge, MA), DMP1 (polyclonal antibody produced at UIC), TRIP-1 (sc374156, Santa Cruz Biotechnology, Dallas, TX), GRP78 (sc166490, Santa Cruz Biotechnology, Dallas, TX), and FN (F3648, Sigma Aldrich) antibodies in 1% BSA-PBS at 4°C overnight. Following PBS washing, the sections were incubated for 1 hour at room temperature with goat anti-rabbit secondary antibody Alexa Fluor 594 (A11012, Invitrogen) and goat anti-mouse secondary antibody Alexa Fluor 488 (A11029, Invitrogen). A Zeiss confocal microscope at RRC of UIC was used to take pictures of the PBS-washed sections after they had been mounted using Antifade Mounting Medium with DAPI (H-1200-10, Vector Laboratories).

### RT-PCR

The sorted cells of both positive CD31^+^/CD144^+^ and negative fraction CD31^−^/CD144^−^, samples were seeded in the 6 well plates and grown till it reaches 80% confluency. The Invitrogen TRIzol Reagent was used to extract total RNA, which was then transformed into cDNA. Real-time PCR was carried out in compliance with the published protocol. (Ramachandran et al., 2016). The gene expression levels were quantified using the 2^−ΔΔCT^ approach, with the GAPDH gene expression level serving as an internal control. Primers were manufactured by IDT (Integrated DNA Technologies, Inc.) and are listed in Table1.

**TABLE 1:** Primer sequence used for real-time PCR targeting endothelial differentiation markers, with GADPH serving as the endogenous housekeeping gene reference.

*Genes*	*Forward Primer (5′-3′)*	*Reverse Primer (5′-3′)*
*Angiopoietin 1*	*AGC GCC GAA GTC CAG AAA AC*	*TAC TCT CAC GAC AGT TGC CAT*
*Angiopoietin 2*	*AAC TTT CGG AAG AGC ATG GAC*	*CGA GTC ATC GTA TTC GAG CGG*
*CD31*	*CTG CCA GTC CGA AAA TGG AAC*	*CTT CAT CCA CCG GGG CTA TC*
*Endoglin*	*TGC ACT TGG CCT ACA ATT CCA*	*AGC TGC CCA CTC AAG GAT CT*
*VE-Cadherin*	*TTG GAA CCA GAT GCA CAT TGA T*	*TCT TGC GAC TCA CGC TTG AC*
*VEGFA*	*AGG GCA GAA TCA TCA CGA AGT*	*AGG GTC TCG ATT GGA TGG CA*
*GAPDH*	*GGA GCG AGA TCC CTC CAA AAT*	*GGC TGT TGT CAT ACT TCT CAT GG*

### *In vitro* tubule assay

The sorted positive fraction of CD31^+^/CD144^+^, along with the negative fraction CD31^−^/CD144^−^, were cultured in EBM2 media for 2 days. A µ-slide angiogenesis glass bottom plate was coated with 10 µL of Matrigel (BD Biosciences, San Jose, CA) and incubated at 37°C for 30 min. Following this, 15k sorted cells/well were seeded onto the Matrigel-coated plate and incubated at 37°C. Tubular structure formation was noted at several time points (2, 4, and 24 h) using an inverted fluorescence microscope (Zeiss Observer D1). Quantitative measurements of tube length, number of nodes, number of mesh, and segments were performed using the “Tube Formation FastTrack AI Image Analysis” (ImageJ).

### Immunofluorescence

Tubule formed cells in the µ-slide angiogenesis glass bottom plate was confirmed through the microscope and the media was carefully removed without causing any damage to the tubule structure. Simultaneously, the unsorted DMP1 treated DPSCs were grown on a cover glass for 7 days. Following that, the tubule-formed cells and the cells grown on the cover glass were fixed with 10% neutral formalin for an hour at 4°C. They were then rinsed with PBS and permeabilized with 0.1% Triton X-100 in PBS for 10 min at room temperature. This was followed by blocking with 5% BSA in PBS for 1 h. Primary antibodies against CD31 (ab28364, Abcam, Cambridge, MA), vWF (SC 365712, Santa Cruz Biotechnology, Dallas, TX), and FN (F3648, Sigma Aldrich), were added and incubated for overnight at 4°C. Following, the addition of fluorescently labelled secondary antibodies (goat anti-rabbit Alexa Fluor 594 (A11012, Invitrogen), goat anti-mouse Alexa Fluor 488 (A11029, Invitrogen), were incubated for 2 h at room temperature. Finally, the tubule was stained with DAPI for nuclear staining for 5 mt. The tubules were then imaged at the UIC RRC Facility using a Zeiss 710 Meta Confocal Microscope.

### Statistical analysis

Data are presented as the mean ± standard deviation of at least three independent experiments. Statistical significance was assessed utilizing the Student’s t-test. Significance was attributed to p-values of ≤0.05 and ≤0.01.

## Results

### Endothelial cells differentiated from DPSCs by flow cytometry cell sorting

**FIGURE 1:**
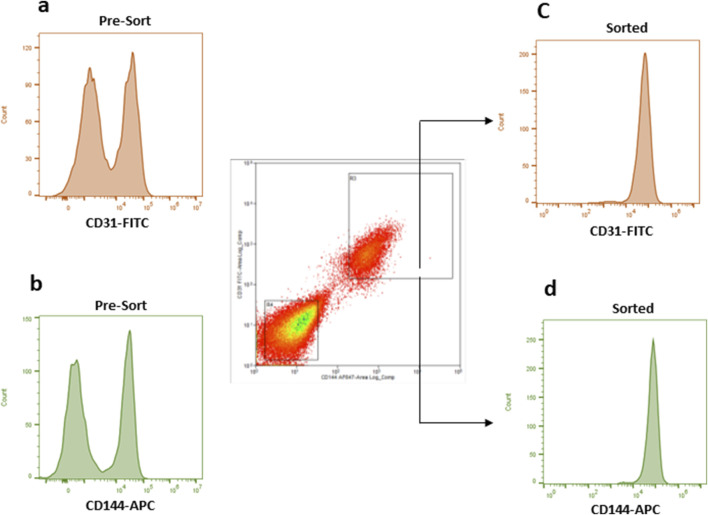
The flow data of the unsorted and sorted DMP1 treated DPSCs. Flow cytometry was used to analyze CD31 and CD144 expression in DMP1-treated DPSCs before **(a,b)** and after sorting (1c and 1d). The cells were stained with antibodies against CD31 (FITC) and CD144 (APC), using FITC or APC labelled isotype match IgG as control antibodies to set the correct gating. After 7 days, DMP1 treated DPSCs were sorted based on positive CD31 and CD144 antibodies. The cells that were positive for both markers, CD31 and CD144, were considered as endothelial cells **(c,d)**. Whereas the negative group did not express these markers.

To obtain a potentially functional population of ECs, DPSCs cultured in HUVEC-ECM supplemented with DMP1 for 7 days were initially assessed for the expression of the endothelial markers CD31 and CD144. In this unsorted population, flow cytometry showed that approximately 46% of the cells expressed CD31 (Figure 1a), and 44% expressed CD144 (Figure 1b). To enrich for cells exhibiting endothelial traits, the mixed population was subsequently subjected to fluorescence-activated cell sorting. During sorting, cells co-expressing CD31^+^ and CD144^+^ were isolated as the positive fraction, achieving a high purity of around 95% (Figures 1c,d). In contrast, cells lacking both markers were collected as the negative fraction, representing the non-endothelial-like population.

### Transcriptional changes of key endothelial markers in the sorted and unsorted DMP1 treated DPSCs

**FIGURE 2:**
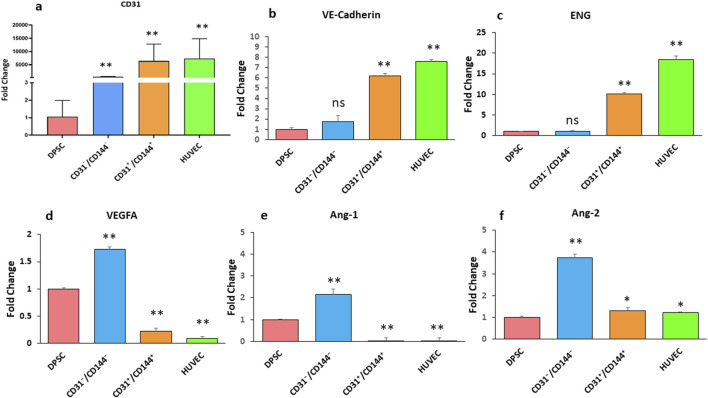
Expression of endothelial-specific markers in DPSCs derived endothelial cells. RT-PCR analysis was performed to assess the expression levels of CD31 **(a)**, VE-cadherin **(b)**, ENG **(c)**, VEGFA **(d)**, Ang-1 **(e)**, and Ang-2 **(f)** in two distinct cell populations: a positive fraction enriched for CD31^+^/CD144^+^ endothelial cells, and a negative fraction lacking these markers (CD31^−^/CD144^−^). Gene expression results are presented as fold changes relative to unstimulated DPSCs. * Indicates p < 0.05 and ** indicates p < 0.01.

Expression of angiogenic specific markers, including CD31 (Figure 2a), VE-cadherin (Figure 2b), Endoglin (ENG) (Figure 2c), VEGFA (Figure 2d) Angiopoietin-1 (Ang-1) (Figure 2e), and Angiopoietin-2 (Ang-2) (Figure 2f), were observed in the sorted and unsorted population as indicated in Figure 2. Interestingly, compared to the negative fraction and DPSC unsorted cells, the CD31^+^/CD144^+^ positive population had higher expression of CD31, VE-Cadherin, and ENG transcripts. Remarkably, this angiogenic gene expression is possibly similar to HUVEC cells. However, DPSCs sorted negative fraction had noticeably high gene expression of VEGFA, Ang-1, and Ang-2.

### Evaluation of angiogenic function of sorted CD31^+^/CD144^+^ and CD31^−^/CD144^−^ cells, by capillary-like tube formation assay

**FIGURE 3:**
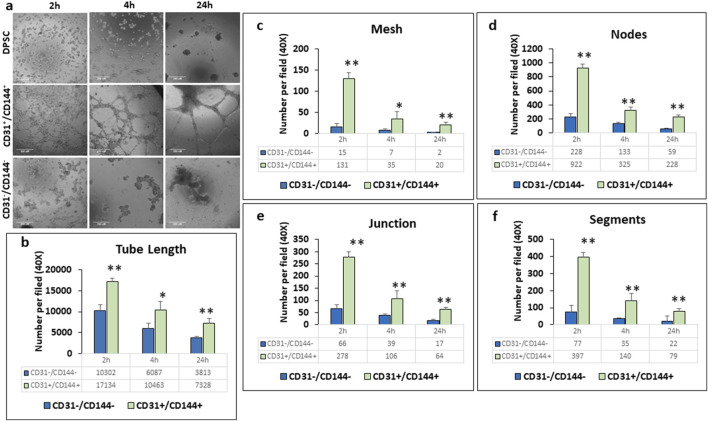
CD31^+^/CD144^+^ positive cells exhibited greater tubule formation compared to CD31^−^/CD144^−^ negative cells. Endothelial-like cells, sorted from DPSC-derived populations treated with HUVEC-ECM in the presence of DMP1, were seeded onto plates coated with growth factor-reduced Matrigel. Representative photomicrographs **(a)** show that the CD31^+^/CD144^+^ positive cell fraction formed more extensive tubule-like networks than the CD31^−^/CD144^-^ negative fraction. Scale Bar = 250 μM. Measurements related to tubule formation in the negative (blue bars) and positive (green bars) fractions of the sorted cells include tube length **(b)**, mesh area **(c)**, number of nodes **(d)**, junctions **(e)**, and segments **(f)**. Mean and SD from three measurements are presented. *: p < 0.05 and **: p < 0.01.

We then examined the sorted ECs for its function as typical vascular ECs by assessing their ability to form capillary-like tubes. Results in Figure 3a showed higher number of capillary-like tubules in the CD31^+^/CD144^+^ sorted population. Within 2–4 h, capillary-like tubules formed into organized tubular networks, demonstrating a significant enhancement in branching and vascular network formation. Quantitative analysis revealed a significant increase in tube characteristics, including length (Figure 3b), Mesh (Figure 3c), Nodes (Figure 3d), Junction (Figure 3e), and segments (Figure 3f). In contrast, the negative fraction, lacking ECs, exhibited cellular aggregates with sporadic network formation, followed by tubule aggregation at 24 h. Similar changes were observed in untreated DPSCs.

**FIGURE 4:**
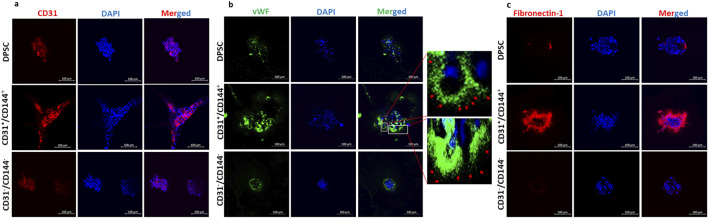
The expression of endothelial-specific markers in well-formed tubules of the CD31^+^/CD144^+^ positive fraction of the DMP1 treated DPSCs: The expression of endothelial-specific markers CD31, vWF, and fibronectin-1 was evaluated in the sorted positive and negative fractions. CD31 **(a)**, vWF with rod-shaped WPBs expression **(b)**, and fibronectin **(c)** were highly expressed in the sorted positive fraction compared to the negative and control DPSCs. The nuclei were stained with DAPI (blue). The scale bar in the images represents 100 µm. Immunofluorescence images show vWF staining in green and nuclei in blue. These images reveal rod-shaped organelles resembling WPBs in DPSCs treated with DMP1. In contrast, the control DPSCs exhibited either shorter WPBs or a complete absence of these organelles compared to those observed in the DMP1-treated group. The scale bar in the images represents 50 μm.

Immunocytochemistry was performed to demonstrate the presence of EC markers in the sorted endothelial cell population. CD31 (Figure 4a) and vWF (Figure 4b) were strongly expressed in the tubes and nodules formed by the CD31^+^/CD144^+^ ECs when compared with the parent DPSCs and the CD31-/CD144-negative fraction. vWF is often stored in Weibel-Palade Bodies (WPBs), that resemble a rod-shaped structure in differentiated ECs. The presence of this secretory organelle was evident in DMP1 stimulated DPSCs in 2-D culture (Supplementary Figure S1). Although CD31^+^/CD144^+^ cell populations show vWF expression staining in the long capillary tubule structures (Figure 4b), the storage of vWF was not clearly discerned in the 3D matrigel culture due to limitations in the light penetration through the thick gel, however, WPBs were present in the capillary like structures. Additionally, the sorted ECs exhibited an increase in fibronectin expression in the spheroids and sprouts (Figure 4c). Fibronectin was absent in the CD31-/CD144-negative fraction DPSCs, whereas the control DPSCs show slight expression, likely due to the heterogenous nature of the cell population.

### *In vivo* evaluation of vasculogenesis in the sorted and unsorted DMP1 treated DPSCs

**FIGURE 5:**
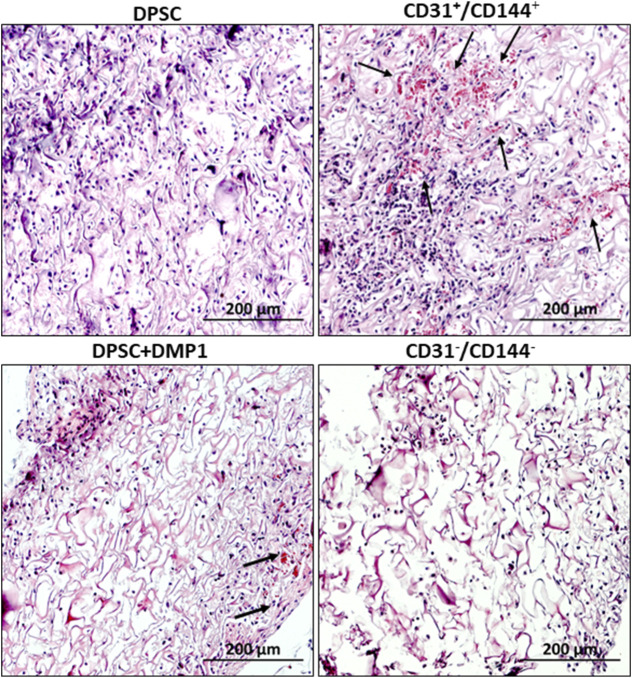
Tissue architecture of subcutaneous explants. Neo-vascularization within the implanted scaffold was observed on day 14 in tissue sections from nude mice, as determined by H&E staining. The endothelial-sorted fraction derived from DPSCs, primarily composed of CD31^+^/CD144^+^ positive cells, exhibited greater neo-vascularization compared to unsorted DPSC cells treated with DMP1 alone as well control DPSCs and CD31^−^/CD144^−^ negative cells. The blood vessel formation is shown by the arrow mark. Scale bar = 200 μM.

**FIGURE 6:**
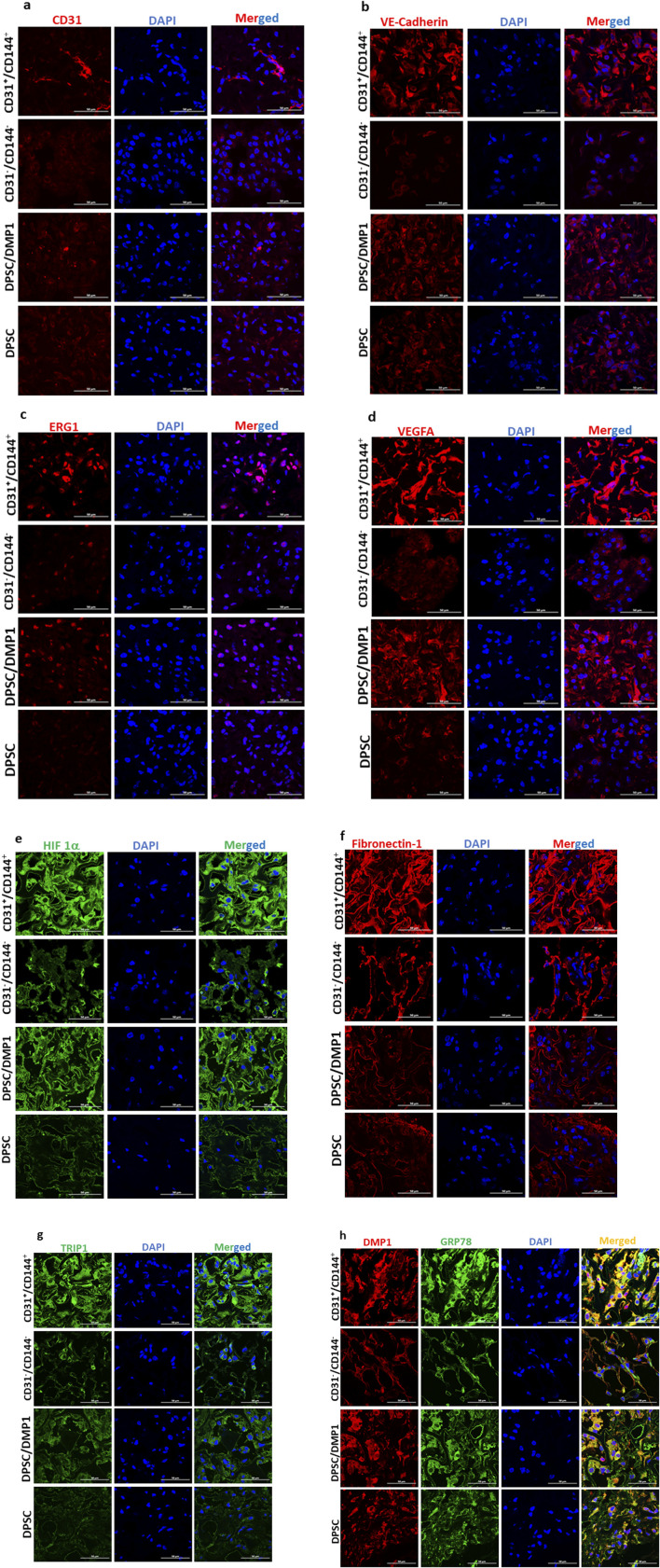
Positive fraction of CD31^+^/CD144^+^ cells show increased vasculogenic effect in *in vivo* implants. CD31^+^/CD144^+^ positive and CD31^-^/CD144^-^ negative fraction were seeded in biodegradable scaffolds and transplanted into the subcutaneous space of immunodeficient nude mice. The scaffolds were removed, fixed, and paraffin embedded 2 weeks following transplantation. Day 14 implanted tissue sections were subjected to immunohistochemical staining for CD31 **(a)**, VE-cadherin **(b)**, ERG **(c)**, VEGFA **(d)**, HIF1-α **(e)**, Fibronectin **(f)**, TRIP-1 **(g)**, and DMP1/GRP78 **(h)**. Images were captured using confocal microscopy and representative images are presented. DAPI (blue) was used to stain the nuclei. 50 μm is the scale bar.

(a) Histological Evaluation: The *in vivo* potential of the sorted ECs to promote vaculogenesis was assessed. To evaluate whether the sorted endothelial like cells obtained from the DPSCs maintain their angiogenic properties, we examined the explants obtained from control DPSCs, unsorted DPSCs treated with DMP1, sorted CD31^+^/CD144^+^ positive cells and CD31^-^/CD144^−^ negative cells. Histological analysis (Figure 5) showed robust vascularization and connective tissue deposition in the tissue sections obtained from CD31^+^/CD144^+^ sorted cells and DMP1-treated DPSCs. The arrows in the images point to visible blood vessels with red blood corpuscles present as a result of active blood flow and successful anastomosis between the newly formed vasculature and the host’s blood vessels. Limited blood vessels were observed with DPSC cells while CD31^−^/CD144^−^ DPSCs was avascular in nature. Consistent with *in vitro* findings, the positive fraction cells demonstrated greater vasculogenic activity when compared with the tissues from the other 3 groups of DPSCs.
(b) Immunohistochemical Evaluation: To further validate the presence of endothelial markers in the CD31^+^/CD144^+^ sorted cells, Immunohistochemistry (IHC) was performed. Explant sections were immunostained with antibodies for endothelial markers CD31, VE-cadherin, and ERG. As shown in Figures 6a–c, the regenerated tissues in both unsorted and sorted DMP1 treated DPSCS groups displayed blood vessels positive for human CD31, VE-cadherin, and ERG. Notably, using a human-specific CD31 antibody, specific to human ECs, we found that scaffolds seeded with CD31^+^/CD144^+^ cells formed approximately twice as many blood vessels as those seeded with unsorted DMP1-treated DPSCs. In contrast, the negative fraction, which lacked ECs, showed a complete absence of blood vessels. This indicates the propensity of purely homogenous population of CD31^+^/CD144^+^ to promote angiogenesis. Additionally, elevated expression of VEGFA (Figure 6d) and HIF1α (Figure 6e) was observed in CD31^+^/CD144^+^ DPSCs. IHC analysis also revealed increased levels of ECM proteins that promote angiogenesis through endothelial cell activation. As shown in Figures 6f–h, CD31^+^/CD144^+^ positive cells exhibited higher expression of fibronectin compared to both the unsorted DMP1 treated DPSCs and the sorted negative fraction. Similar expression patterns were seen for TRIP1 (TGF-β receptor type II-interacting protein), DMP1, and GRP-78 (glucose-regulated protein 78), all known ECM proteins that support vasculogenesis.

## Discussion

The development of functional vasculature is crucial to the success of most tissue engineering applications. ECs play key role in delivering oxygen and nutrients, modulating immune cell traffic and maintaining tissue homeostasis (Trimm and Red-Horse, 2023). Thus, there is a clinical need for production of ECs, engineered vascular tissue grafts and ECs-based cell therapy for the development of vascularization therapeutics.

Due to their notable ability to multilineage differentiate into endodermal, mesodermal, and ectodermal tissue, DPSCs are considered as a promising source of stem cells for regenerative medicine (Mattei et al., 2021). It is still unknown, though, if all DPSCs are naturally multipotent or if they are a diverse population with unique clones, expression markers, and proliferative and differentiating capacities. (Martellucci et al., 2020).

Our study has uncovered a significant role of DMP1, a protein previously implicated in the odontogenic differentiation of DPSCs, in transforming DPSCs into vasculogenic ECs. DMP1 is an extracellular matrix regulatory protein that functions in matrix mineralization. We had earlier demonstrated that DMP1 along with biological cues from the HUVEC-ECM is responsible for transforming DPSCs into vasculogenic ECs (Ganapathy et al., 2024). The findings adds to the earlier research that DPSCs has regenerative potential in differentiating into different lineages (Grosso et al., 2017), Interestingly, it also finds rise in newer applications in bone and neural regeneration in the oral and maxillofacial areas (Fujii et al., 2023). Furthermore, the differentiation of DPSCs into corneal endothelial-like cells (Bosch et al., 2021), supports the idea that DPSCs have significant flexibility towards endothelial lineages. Based on the above findings by other groups, our findings show a unique combination of DMP1 and HUVEC-ECM that effectively directs endothelial lineage commitment. This enhances the potential of DPSCs in vascular regenerative applications. We used HUVEC-ECM and DMP1 stimulation to direct DPSCs to differentiate into *de novo* endothelial-like cells with angiogenic characteristics of ECs because DPSCs are heterogeneous in nature. Since CD31^+^ and CD144^+^ antibodies are both recognized indicators for ECs, they were utilized in fluorescence-activated cell sorting to separate the undifferentiated and differentiated DPSCs. The ability of the sorted CD31^+^/CD144^+^ and CD31^−^/CD144^−^ cells to express endothelial transcripts, act as ECs, and validate their phenotype *in vivo* were all investigated in this study.

Our results demonstrate that CD31 expression was found in about 40% of the sorted population demonstrating that DMP1 had the tendency to transform DPSCs into endothelial lineage. Gene expression analysis of the sorted CD31^+^/CD144^+^ population provided evidence that this endothelial-like cell population maintains the angiogenic properties of HUVECs cells, while CD31^-^/CD144^-^ negative fraction did not. Notably, we discovered that DMP1 stimulation markedly increased the expression of CD31, VE-Cadherin, and Endoglin (ENG) in CD31^+^/CD144^+^ population. CD31 is a cell adhesion molecule present on all ECs and play an important role in angiogenesis and vascular permeability. Previous studies have shown that the expression of ENG is high in DPSCs thereby showing their angiogenic potential (Nakashima et al., 2009). Studies also (Liu et al., 2014) showed that ENG is required for the stem cell derived ECs to organize effectively into tubular structures. This report is consistent with our findings.

Similarly, VE-Cadherin gene expression in the positive fraction samples closely resembled that of HUVEC-positive cells. However, the expression of Ang-1 and Ang-2 was low in the positive fraction, while it is high in the negative fraction. Ang-1 and Ang-2 have opposing roles in vessel development. Ang-1, a natural inhibitor of vascular permeability, helps prevent plasma leakage, whereas Ang-2 contributes to vessel destabilization by detaching smooth muscle cells and promoting increased permeability (Maisonpierre et al., 1997; Thurston et al., 2000). Moreover, their expression is not a universal feature of all endothelial cells and are expressed *in vivo* in vascular remodeling and inflammation. This study also confirms that ECs by itself do not typically secrete VEGFA and this is confirmed by its absence in the endothelial-like cells of the positive fraction similar to HUVECS. However, VEGFA expression is higher in the negative fraction, which consists of undifferentiated DPSCs. The secreted VEGFA binds to VEGFR1 on DPSCs, in an autocrine manner directing these cells toward endothelial differentiation (Bergamo et al., 2021). Meanwhile, the VEGFA released by DPSCs can function in a paracrine manner by binding to VEGFR2 on ECs, initiating angiogenic signaling pathways that promote vasculogenesis (Abhinand et al., 2016; Wang et al., 2020).

A characteristic feature that shows the ECs functionality is the formation of vascular tubules in a three-dimensional environment. In this study, the CD31^+^/CD144^+^ population spontaneously assembled into capillary like structures that resembled tubes. Conversely, the negative fraction, which lacks CD31^+^/CD144^+^ expression developed aggregate-like structures. To confirm the vascular phenotype of the tube-like structures generated by CD31^+^/CD144^+^ cells, *in situ* immunostaining revealed the expression of endothelial markers CD31, vWF, and fibronectin, validating the presence of ECs. Specifically, vWF is stored in WPBs, a specialized multimeric tubular secretory organelle unique to mature ECs (Lenting et al., 2015; Yadegari et al., 2022). Our study revealed strong vWF staining in CD31^+^/CD144^+^ cell populations, whereas the CD31^-^/CD144^-^ population showed minimal expression. Previous published findings from our group have shown the vWF expression along with WPBs at 7 and 14 days of DMP1 treated DPSCs (Ganapathy et al., 2024). This observation provides a more precise interpretation of our results and aligns with previous studies showing clear examples of WPBs imaging using immunofluorescence (Yadegari et al., 2022)

*In vivo* evaluation of the vasculogenic potential demonstrated the presence of numerous human vascular networks containing erythrocytes in the matrix of the CD31^+^/CD144^+^ population confirming that the sorted ECs maintained their angiogenic phenotype *in vivo*. ERG1, CD31, VEGFA, VE-Cadherin, FN1, HIF1α, and GRP78 all showed positive staining in the explants. Interestingly, we found that DMP1 significantly raised VEGFA expression in both DPSCs stimulated with DMP1 and sorted ECs. Through the transcription factor ERG, VEGFA can increase the expression of VE-Cadherin. These results align with the findings of (Sasaki et al., 2020). The endothelial transcription factor ERG is necessary to preserve endothelial homeostasis, and it was found to be expressed in both the tubule-like structures formed by the sorted ECs and the *in vivo* explant sections. Prior research has demonstrated that adult mice with endothelial ERG deletion have impaired angiogenesis, decreased endothelial homeostasis, and increased tissue inflammation. (Birdsey et al., 2015).

Additionally, VE-Cadherin was also detected in *in vivo*. Notably, CD31^+^/CD144^+^ population exhibited the increased expression of hypoxia-inducible factor 1-alpha (HIF-1α). Since, it is well known that HIF-1α plays a critical role in upregulating VEGFA, a key regulator factor essential for the angiogenic phenotype observed in these transformed DPSCs thereby facilitating neovascularization (Zimna and Kurpisz, 2015). In contrast, the lower levels of HIF-1α and VEGF in the unsorted DPSCs treated with DMP1 was observed. Further, the presence of HIF-1α in the endothelial like positive population supports their angiogenic potential. The first stage of inflammation during intramembranous bone regeneration creates a hypoxic environment that encourages osteoblasts to release pro-angiogenic factors, such as VEGF, via the HIF-1α pathway. This promotes EC migration, proliferation, and increased vascular permeability. (Wang et al., 2007).

Fibronectin polymerization is known to be crucial for the survival, growth, and tube formation of ECs. (Wijelath et al., 2004). Mice with knockout of the fibronectin splice variants EIIIA and EIIIB exhibit significant vascular defects (Hensel et al., 2021). According to the report, fibronectin’s interaction with VEGFA demonstrates that it is a crucial determinant of angiogenic activity. (Usuelli et al., 2021). Fibronectin in the matrix of sorted ECs suggest its role in angiogenesis. TRIP1 was identified in the dentin matrix and plays a crucial role in organizing the ECM, promotes angiogenesis by aiding the secretion of extracellular vesicles, and supports the synthesis of important osteogenic ECM proteins (Chen and George, 2018). Previous findings from our lab have shown that GRP78 overexpression stimulates the expression of osteogenic and angiogenic markers in an *in vivo* subcutaneous implant model (Merkel et al., 2023). GRP78 was identified as a membrane receptor for DMP1. Higher expression of GRP78 and DMP1 suggests the involvement of both the receptor and ligand in vasculogenesis.

The data presented here demonstrate that DMP1 stimulus along with biological cues provided by HUVEC ECM transformed 40% of the DPSCs into vascular ECs. The characterization and functionality of the transformed ECs *in vitro* and *in vivo* demonstrate the presence of endothelial markers such as CD31 and vWF and its ability to form tubular structures lined by ECs. Altogether, these results pave the way for therapeutic use of DMP1 in developing therapies to promote vasculogenesis.

## Conclusion

In the current study, results show that DMP1 stimulus along with cues provided by HUVEC-ECM transformed DPSCs into vascular ECs when implanted in mice subcutaneously. To confirm the vasculogenic potential of *de novo* transformed ECs, we performed immunohistochemistry on tissue sections from the explants isolated at 2 weeks post-implantation. Conventional endothelial markers, including CD31, VE-cadherin, ERG, VEGF, Fibronectin, TRIP-1, DMP1 and GRP78 were used to characterize the EC phenotype. At specified time points during the transformation process, we observed increased expression of these markers in the positive fraction of CD31^+^/CD144^+^ cells compared to the negative fraction CD31^−^/CD144^−^ cells indicating that the DMP1 stimulated DPSCs when implanted had successfully transformed into vasculogenic endothelial-like cells.

## Data Availability

The data that support the findings of this study are available from the corresponding author upon reasonable request.

## Supplementary material

SUPPLEMENTARY FIGURE S1 Immunofluorescence images show vWF staining in green and nuclei in blue. These images reveal rod-shaped organelles resembling WPBs in DPSCs treated with DMP1. In contrast, the control DPSCs exhibited either shorter WPBs or a complete absence of these organelles compared to those observed in the DMP1-treated group. The scale bar in the images represents 50 μm.
