# Supplementary figures and images for: DMP1-mediated transformation of DPSCs to CD31+/CD144+ cells demonstrate endothelial phenotype both in vitro and in vivo
Source: Front Cell Dev Biol. 2025 Aug 8;13:1630129. doi: 10.3389/fcell.2025.1630129 (PMC12371194; doi:10.3389/fcell.2025.1630129)

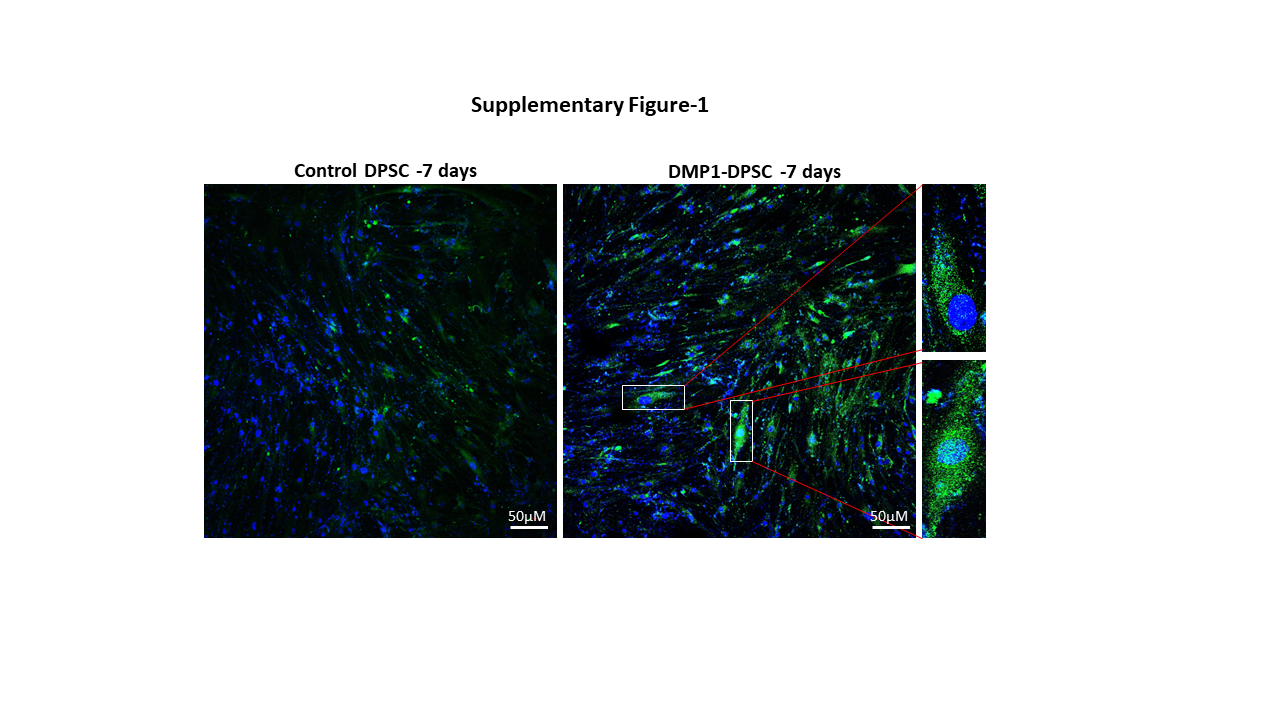

Supplement: Supplementary file 1 [file Image1.tif]
